# A Lattice Model for Influenza Spreading

**DOI:** 10.1371/journal.pone.0063935

**Published:** 2013-05-22

**Authors:** Antonella Liccardo, Annalisa Fierro

**Affiliations:** 1 Physics Department, Università degli Studi di Napoli “Federico II”, Napoli, Italy; 2 Istituto Nazionale Fisica Nucleare (INFN) - Sezione di Napoli, Napoli, Italy; 3 Consiglio Nazionale delle Ricerche (CNR) - Institute Superconductors, Oxides and Other Innovative Materials and Devices (SPIN), Napoli, Italy; University of Zaragoza, Spain

## Abstract

We construct a stochastic SIR model for influenza spreading on a D-dimensional lattice, which represents the dynamic contact network of individuals. An age distributed population is placed on the lattice and moves on it. The displacement from a site to a nearest neighbor empty site, allows individuals to change the number and identities of their contacts. The dynamics on the lattice is governed by an attractive interaction between individuals belonging to the same age-class. The parameters, which regulate the pattern dynamics, are fixed fitting the data on the age-dependent daily contact numbers, furnished by the Polymod survey. A simple SIR transmission model with a nearest neighbors interaction and some very basic adaptive mobility restrictions complete the model. The model is validated against the age-distributed Italian epidemiological data for the influenza A(H1N1) during the 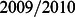 season, with sensible predictions for the epidemiological parameters. For an appropriate topology of the lattice, we find that, whenever the accordance between the contact patterns of the model and the Polymod data is satisfactory, there is a good agreement between the numerical and the experimental epidemiological data. This result shows how rich is the information encoded in the average contact patterns of individuals, with respect to the analysis of the epidemic spreading of an infectious disease.

## Introduction

There are two major approaches to model the spreading of an infectious disease in a space-structured population that are mostly used in recent literature: the Individual Based Models (IBM) and the Metapopualtion Models. The first ones [1]–[4] are obtained by coupling a transmission model with a highly detailed socio-demographic model, based on a thorough knowledge of the population structure and its distribution in age-classes, household size, school size, employments, etc. An appropriate set of social contact groups is associated to each individual according to her/his age. The epidemiological state of any individual is updated at each time step, typically assuming a homogeneous mixing within each context. The Metapopulation models [5]–[12] are mainly focused on the role of human mobility, and involve a very accurate knowledge of the mobility fluxes between different sub-populations (transportation infrastructures, long-range airline connections, short range daily commuting pattern, etc.). In each sub-population, the disease typically propagates according to a discrete stochastic model with homogeneous mixing. Both these large-scale approaches are extremely fascinating, realistic and predictive. Comparative studies also prove the two approaches to be in good agreement with each other [13]. However, the price to pay for the realism of these models is the high computational cost and their non trivial generalizability, due to the huge amount of input data necessary to achieve a realistic description of the populations living in different geographic areas, and/or their connections at global level.

In the present paper, we follow a complementary approach, in which we reduce the number of parameters and input data as much as possible. In particular, we concentrate on a few key factors, assumed to be relevant for the spreading of an infectious disease, and construct a simple model, which is able to reproduce the age-dependent epidemiological curves of an epidemic outbreak, as for instance the H1N1 pandemic in Italy in the season 2009/2010.

The ingredients that we include in our model are the following:

- *Age-Classes.*

The distribution in age-classes is generally acknowledged to be a crucial feature for the spreading of an infectious disease [14], [15]. Individuals of different ages have different pre-existing partial immunity, hygienic habits and mobility patterns which, in turn, makes the susceptibility to be age-dependent. In our model, we consider a population that is age distributed in 4 age classes (0–4, 5–14, 15–64, over 65 years old), which are the age-classes typically used in the public health data report on influenza spreading.

- *Heterogeneous and dynamic nature of contacts among individuals.*

The hypothesis of homogeneous mixing does not take properly into account the complexity of human interactions. Individuals typically have contacts with a finite number of other individuals and the number and identity of contacts change in time. Epidemic models formulated on networks naturally include heterogeneity. In these models, the disease transmission network is drawn over the underlying network of contacts (i.e. the socio-demographic model). The literature on the epidemic spreading on networks is extensive (e.g. [16]–[22]) and it mostly consists of models formulated on static networks. Dynamic contacts are instead allowed in the neighbor exchange model of Ref. [23], where each individual has a fixed number of contacts, but the identities of contacts change in time. In Ref. [24], susceptibles can avoid contacts with infected individuals by rewiring their connections and changing the identity of their contacts. This is an example of adaptive behavior where the dynamics of contacts is induced by the epidemic itself. The epidemic spreading on dynamic small world networks is also discussed in Refs. [25]–[27]. Comparative studies on the effect of dynamics on small world network [28] show that the risk of an outbreak on dynamic network is higher than in static models.

In our model, the network of contacts is a simple *D*-dimensional regular lattice with a certain percentage of empty sites. The use of lattice models is not a novelty in the literature on epidemic models. For instance, in Ref. [29], a SIRS model is formulated on a regular lattice, in which, each site is occupied by an individual, and all the individuals have the same number of contacts, with fixed identity. The dynamical variables of the model are those related to the infection, and change according to the state of the nearest neighbors. In our model, instead, the existence of empty sites allows heterogeneity in the number of contacts. Furthermore individuals change the number and identities of their contacts, by moving from a site to a nearest neighbor empty site according to certain mobility rules.

- *Balance between random nature of contacts and assortativity.*

Data on the age-dependent contact patterns are crucial to study the spreading of close-contacts infectious diseases. This is the basic idea of the *social contact hypothesis*, which assumes that the age specific number of potentially infectious contacts is proportional to the self reported number of social contacts in each age-class [30]. In the Polymod project [31], a diary-based large scale survey of epidemiologically relevant contact patterns was performed in 8 EU countries. The country dependent contact matrices, 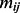 (i.e. the average number of daily contacts that an individual in the age-class *j* has with individuals in the age-class *i*) were obtained from the self reported number of social contacts in each age-class. The dominant feature of these contact matrices, all over Europe, is the large diagonal elements (larger in the 5–24 age-class than in the adult ones), which indicate that individuals prefer contacts with individuals belonging to the same age-class, i.e they have an assortative behavior. A strong assortative behavior is also found in Ref. [32], where authors generate synthetic contact matrices through an IBM model, by coupling information obtained from the Italian time-use data (carried out by the ISTAT - www.istat.it), and socio-demographic data, and compare the results with questionnaire-based contact matrices.

Starting from the self reported social contacts of the Polymod survey, the age specific transmission rates may be estimated [30], [31], [33]. The focus on time-use data is instead at the base of Ref. [34], where authors make use of time-use surveys data to measure contact patterns and to explain observed seroprevalence profiles.

In our model, we also make the assumption that the spreading of an infectious disease is mainly regulated by the average age-dependent contact patterns of individuals, and design a dynamic model with the aim to reproduce those patterns. In particular, the dynamics of individuals is governed by a lattice-gas inspired Hamiltonian, in which individuals of the same age-class interact via an attractive potential. The effect of this potential is to favor energetically those configurations, in which nearest neighbor sites are occupied by individuals belonging to the same age-class, thus introducing a tendency to assortativity. The parameters, which govern the dynamic behavior of individuals, are chosen to reproduce the total daily number of contacts of Polymod data in each age class.

- *Adaptivity.*

The spreading of an epidemic induces modifications of the human behavior such as restrictions of mobility and contacts. These behavioral changes modify the underlying (disease independent) dynamic network, which becomes adaptive to the disease [23]. The effect is more pronounced in the case of lethal diseases, or whenever the spreading of the epidemic is accompanied by the spreading of awareness and fear. At the same time, the behavioral changes, which occur as a consequence of the epidemic spreading, affect the evolution of the epidemic itself [35], [36].

In the present model, we simply introduce some very basic rules of social distancing, which are over-imposed to the usual mobility rules during the epidemic. The upgrade of the model in order to include the effects of self-initiated behavioral changes will be discussed in a forthcoming paper.

Summarizing, the main motivation of the present study is to show that a simple SIR model for the spreading of an infectious disease, coupled with a lattice-gas model for the underlying contact dynamics, is able to reproduce the age-dependent epidemiological curves of an epidemic outbreak. Our crucial hypothesis is that a detailed microscopic knowledge of the population structure, although powerful and fascinating, is not strictly necessary in order to reproduce the spreading of an infectious disease, which is instead essentially regulated by the average contact patterns of individuals. Under this hypothesis, any model, which is able to reproduce such patterns, may in principle predict the epidemics dynamics, if some disease dependent parameters are provided. The focus on the contact patterns is a novelty with respect to Ref. [37], where the parameters controlling the dynamics were fixed a priori, and the daily numbers of contacts were not compared with experimental data. In the present paper, for different lattice topologies, the parameters, which regulate the pattern dynamics, are instead fixed in order to obtain the best agreement with the Polymod data. The socio-demographic model is then coupled with a SIR model with a nearest neighbor interaction for the spreading of the infection. The model is checked on the Italian epidemiological data for the influenza A(H1N1) during the 2009/2010 season. If the accordance between the contact numbers of the model and the Polymod data is satisfactory, a good agreement is also found between the numerical and the experimental epidemiological data.

## The Model

The model is an attractive lattice-gas on a *D*-dimensional lattice, which represents the dynamic contact network of individuals [37]. Individuals move on the lattice according to certain mobility rules designed in order to implement the assortative behavior of different age-classes. The parameters, which regulate the pattern dynamics, are fixed in order to obtain the best agreement with the data on the total age-dependent daily contact numbers, furnished by the Polymod large-scale survey. The transmission model is a SIR model with a nearest neighbor interaction for the spreading of the infection, with the addition of some adaptive mobility restrictions during the epidemic.

### Lattice Structure - The Demographic Model

The demographic structure of the model is very simple: the population is randomly distributed on a *D*-dimensional lattice according to the age group densities of a specific country. In particular the lattice is occupied by *N* individuals of 4 different types, labeled by the index 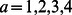 corresponding respectively to the age groups, 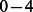, 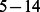, 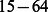 and over 65 years old. The lattice represents the contact network of individuals: contacts and transmission of the infection occur only between nearest neighbors. The dimension *D* fixes the maximum number of simultaneous contacts that an individual can have. Here, we choose to work with a Neumann neighborhood (i.e. with 2*D* nearest neighbors), the extension to the case of a Moore neighborhood being straightforward. Periodic conditions are fixed on the lattice boundary.

In contrast with other lattice models [29], the sites are not all occupied. The existence of empty sites is a crucial feature of the model because individuals change the identities (and eventually the number) of their simultaneous contacts by moving from a site to a nearest neighbor empty site. We remark that, when moving from a site to a nearest neighbor empty site, the individual moves from a certain environment/social group to another (e.g. from work to home), i.e. no notion of distance is defined on the lattice.

### Disease Independent Mobility Rules

In our model, we assume the existence of an underlying dynamic contact network of individuals, which is disease independent. During the epidemic, some very realistic mobility restrictions are over-imposed on the population in order to take into account the adaptive behavior of individuals.

The disease independent network is constructed in order to reproduce the data on the age-dependent daily contact numbers, furnished by the Polymod project [31]. This is a large scale survey performed in 8 EU countries, in which the contact patterns, relevant for infections transmitted by the respiratory or close-contact route, are acquired. As shown in Polymod survey, young people (5–14) have the highest contact rate, followed by adults (15–64), babies (0–4) and old people (over 65). Furthermore, most of the contacts have been observed between persons of similar age.

In details, we define the nearest neighbor effective number, 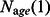, of the individual located at the site 1, and belonging to a certain age-class, as the total number of nearest neighbors belonging to the same age-class. At each step of the dynamics:

1. we randomly choose an individual located at the site 1, and a nearest neighbor destination site, 2, on the lattice. If the site 2 is occupied, another individual is randomly chosen. The probability that the randomly chosen site 2 is empty depends on the local occupation density, increasing as the crowding decreases;
2. if the site 2 is empty, we try to move the individual from the site 1 to the site 2 with the probability:

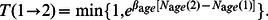
(1)The movement from the site 1 to the site 2 occurs with probability 1 if the number of nearest neighbors in the same age-class increases or remains constant, otherwise it occurs with probability
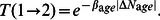
(2)


with 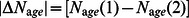


It is worth noticing that the probability that an individual moves from the site 1 to a randomly chosen nearest neighbor site 2 is given by the probability that the site 2 is empty, times 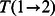, where the first term favors spacing and the second one instead crowding. Notice also that for 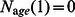 the transition always occurs with probability 1.

The parameter 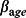 may be considered an age-dependent inverse mobility, and it plays the role of an assortativity regulator. The larger is 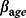, the smaller is the probability that the movement is accepted when 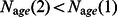: i.e. the larger is 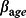 the stronger is the assortativity constraint for the corresponding age-class. For 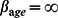, all the movements, which cause a reduction of the number of nearest neighbors in the same age-class, are strictly forbidden. For 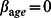, instead, the movement always occurs with probability 1 and we recover the case of purely random diffusion.

Let us emphasize that the efficiency of the assortatitvity constraint does not depend only on 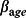, but also on the specific age-class density 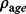, i. e. the same value of 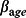 leads to different contacts patterns, when applied to age-classes with different densities. Let us consider for example the case 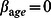 in each age-class. In this circumstance, the distribution probability, 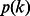, that an individual has 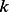 nearest neighbors of her/his own age-class, is simply given by the binomial distribution.
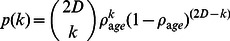
(3)where 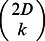 is the binomial coefficient and 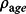 is the occupation percentage of the individuals belonging to that age-class. For each dimension *D*, at low 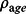, this function is peaked on 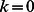, namely the most probable configurations are those with zero nearest neighbors of the same age-class. Under this circumstance, the mobility constraint is quite inefficient. Increasing 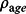 and *D*, the peak moves to value of 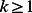, making the constraint on the assortativity more efficient.

The algorithm adopted, Eq.(1), is a standard Metropolis algorithm for a Hamiltonian system in the canonical ensemble. Indeed, Eq. (1) can be derived from the following lattice-gas inspired Hamiltonian.
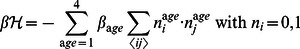
(4)where the sum 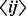 runs over the nearest neighbor site 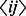, the index 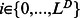 and *L* is the lattice size, 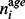 is the age-class occupation number of the site *i*, which is 0, if the site is empty, and 1, if the site is occupied by one individual of the age-class “age”. Notice that each site can be occupied at most by one individual. This Hamiltonian describes an attractive interaction among individuals of the same age-class: the energy of the system decreases when two nearest neighbor sites are occupied by individuals of the same age-class. Individuals of different age-classes interact only by means of the excluded volume.

In the standard Metropolis algorithm, sequential updates of the system are realized, in which only one individual tries to move at each time step. The single particle movement from the site 1 to the site 2 changes the energy of the system as follows
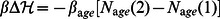
(5)


The transition probability, 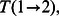 in Eq. (1), satisfies the detailed balance principle, and this assures the equilibrium distribution probability of the system to be the canonical one.

Let us briefly discuss the problem of equilibration in the present model. The system, described in Eq. (4), can be viewed as a mixture of 4 species of particles, with an attractive interaction between particles of the same type. In the case of a single type of particles, it reduces to a lattice-gas model, obtained from a mapping of the Ising model with conserved magnetization. In the Ising model, a second order continuous phase transition, at a finite critical temperature, 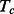, separates a high-temperature disordered paramagnetic phase from a low-temperature ferromagnetic ordered one (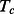 depends on the dimension 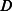 of the lattice). After a quench below the critical temperature, 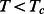, a phenomenon called coarsening is observed. At each instant, domains of up and down spins are present. As time passes, the typical size of the domains increases, however the time needed for a given initial state to reach equilibrium diverges as a function of the system linear size, *L* [38]. In an infinite system, this coarsening process goes on forever.

An analogous phenomenon is observed in the system studied here. At low 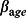 (corresponding to high temperatures), the system is in a homogeneous disordered phase. After a quench at high 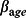 (low temperatures), domains of particles of the same type appear. As time passes, the typical size of the domains slowly increases. The presence of different species, that interact by means of the excluded volume, further complicates the dynamics, since isolated particles in domains of a different type may remain blocked in this state for a long time, although this is not an equilibrium state for the system. We observe that different quenching procedures, in general, produce different final states, for the same set of dynamical parameters, 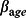, and therefore the final state, although stationary on our observation time scale, may be an out-of equilibrium state.

The Hamiltonian, Eq. (4), with 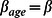 for each *age*, can be also viewed as the Hamiltonian of an annealed site-diluted 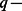
Potts model with 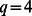 [39], with the constraints that the number of spins in each of the *q* spin states is kept constant. The 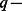
Potts model, which was extensively studied in literature, presents a complex phase diagram with a first or a second order transition depending on the *q* and *D* values. The problem of equilibration using standard algorithm Metropolis with sequential updates of single spin, as the one adopted here, is well known in literature, where cluster [40] or multicanonical algorithm [41] were developed in order to overcome the critical slowing down.

### Transmission Model and Adaptive Mobility Rules

The previous model for the population dynamic must be supplied with an epidemiological model. To this purpose, we construct a SIR stochastic model, with nearest neighbors interaction, in which each individual can be healthy without immunity (i.e. susceptible, S), infective (I) or healthy with immunity (i.e. recovered, R). To describe these possibilities, we associate to each person an internal degree of freedom for the healthy/infective status (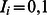), and to healthy individuals we attribute a further degree of freedom for susceptible/immune status (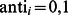). After a potentially contagious contact with an infected nearest neighbor, a susceptible (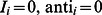) becomes infected (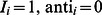) according to her/his specific age-class susceptibility, 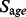. The infective individual goes through an asymptomatic phase, followed by a symptomatic one.

Notice that the transmission probability, 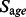, does not coincide with the WAIFW matrix usually considered in literature [42], which indeed represents the probability that an infective of a certain age-class has contact, and therefore infects a susceptible individual of another age-class. In our approach, the dynamics is not encoded into the transmission probability but it is considered separately.

During the epidemic, some disease adaptive rules are over-imposed:

- Infected individuals typically stay at home during the manifestation of symptoms, reducing their contact network essentially to the family. This tendency has been implemented in the model by imposing that, at time *T_S_* (where s stays for stop or symptoms) after the contraction of the infection, the infected individual stops and does not move until she/he recovers.
- Susceptible individuals tend to avoid contacts with the infected ones during their symptomatic phase. This tendency is implemented by imposing that the empty sites that are nearest neighbor to symptomatic infected individuals are interdicted. In other words, symptomatic infected individuals can only infect their susceptible nearest neighbors at the stop time *T_S_*, (i.e. the family in our simplified model).
- The neighbors of infected individuals can move without any restriction.

After a time 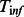 since infection, the infected individual acquires permanent immunity (i.e. develops antibodies), changing the internal d.o.f. 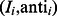 from 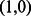 to 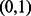, and starting to move again. Each infected individual has her/his own infective period, while the infectivity is taken to be constant during the disease.

We assume the infective period 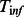 to follow an exponential distribution. This is the most frequent choice in literature and corresponds to assume the recover probability to be independent of the time since infection. More realistic choices for the infective period have been shown to produce a destabilization effect [43]. Different choices for the infected period distribution (e.g. gamma distribution), can be easily implemented in our model. The introduction of a latent and an incubation interval is also straightforward.

## Results

### Contact Dynamics

Applying the dynamics rules described in the previous section, we simulate the contact patterns of individuals in absence of infectious diseases. We perform 3 different numerical experiments (listed in Table 1) on lattices with dimension 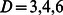 and occupation percentage 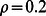. Different values of the linear size of the lattice, 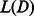, are fixed for different *D*, such that the number of individuals is roughly constant for each dimension (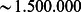 individuals). As reference population, we consider the Italian one, whose age-class distribution is given in Table 2. For each experiment, we simulate 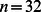 independent processes, and the quantities of interest are obtained averaging over these independent realizations of the system.

**Table 1:** List of Numerical Experiments.

Simulations	D	L	*ρ*	week/MCS
Experiment 1	3	180	0.20	198
Experiment 2	4	50	0.20	148
Experiment 3	6	14	0.20	105

Values of the parameters in the 3 different numerical experiments realized. The linear size of the lattice, *L*, is fixed in order to have roughly the same number of individuals (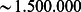) for each dimension, *D*. The conversion factor, necessary to reproduce the dynamics, depends on the lattice topology.

**Table 2:** Age-Class Parameters.

age-group (age)	*f_a_*
0–4	0.048
5–14	0.093
15–64	0.659
+65	0.20

Distribution of the Italian population in age-classes. ISTAT - 2009.

Following [30], we define the contact matrix 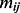 as the average number of contacts that a single individual of age-group *j* has with individuals of age-group *i*. Thus the diagonal term, 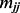, represents the average number of contacts that an individual of age-class *j* has with people belonging to the same age-class, while the quantity 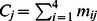, represents the total number of contacts that an individual of age-class *j* has on average in one day with other people, independently of the age-class to which they belong. In the present paper, we fix the parameters 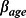 by requiring our simulations to reproduce the total contacts 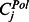 of the Polymod survey, for each of the 4 age-classes considered.

In our simulations, the time unit is the Monte Carlo Step (MCS), that corresponds to the time interval necessary to ensure that each individual attempts to move on average one time. In order to reproduce the Polymod data, we also have to fix the relation between the MCS and the Polymod time unit (i.e. 1 day). The collapse of the numerical data onto the experimental ones in general occurs at different values of the age mobility for different dimensions, and is obtained by suitably fixing the time conversion factor. In particular, the number of MCS corresponding to 1 day reduces, with increasing the dimension of the lattice (see Table 1). This is consistent with the fact that higher dimensional topologies imply a potentially higher number of simultaneous nearest neighbors. As a consequence, in higher dimensions the individual can reach the appropriate number of new contacts expected in one day, with few movements.

For a given set of parameters 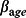, after an initial transient, the system reaches a stationary state, in which the total number of contacts 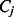 in each age-class *j* is time independent. Notice that, although the number of contacts is not dependent on time on our observation time scale, the final state may be an out-of equilibrium state. Different procedures, in general, may produce different final states, for the same set of dynamical parameters, 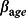. In the present case, this is not relevant since the parameters, 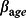, are not observable parameters, the only quantity of interest being instead the contact matrix.

Within each experiment, starting from a state with very small values of 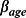, we perform a cooling of the system slowly increasing the parameters 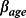 until a small region of the parameter space is reached, in which the total number of contacts is enough close to the experimental data. In such a region, we construct a fine grid in the 4-dimensional parameter space, and evaluate the contact numbers 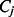 for the 4 age-classes in correspondence to each point of the grid. The final state, adopted to run the epidemic, is chosen as the one which minimizes the 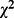 for dependent variables
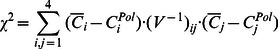
(6)where 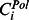 are the total number of contacts obtained with the Polymod survey, 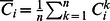 are the simulated number of contacts, obtained as the average of 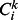 (with 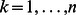) over the *n* independent processes, and *V* is the covariance matrix defined as
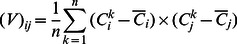
(7)where the sum runs over the n independent processes.

**Figure 1:**
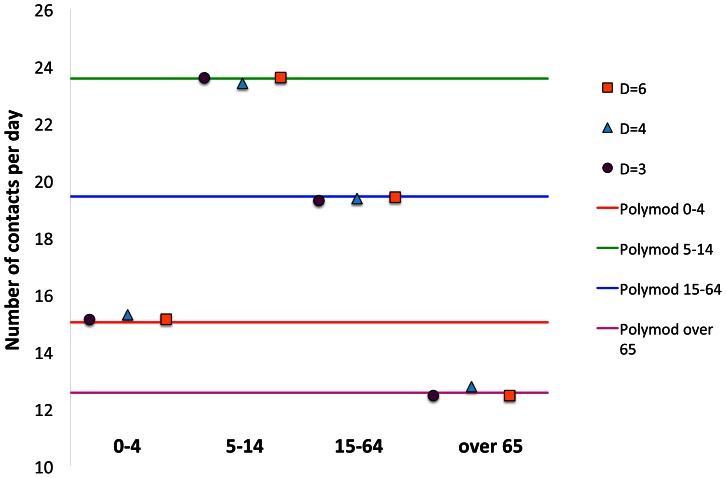
Total number of contacts per day, 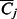, in different age-classes. Comparison between the Polymod data and the simulated data on lattices with different dimensions (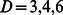).

In Fig. 1, the Polymod data and the simulated values of the total average number of contacts, 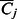, are compared for the 4 age-classes. As shown in Fig. 1, for each value of *D*, our model is able to reproduce the Polymod daily contact numbers, with an appropriate set of parameters, 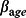.

**Figure 2:**
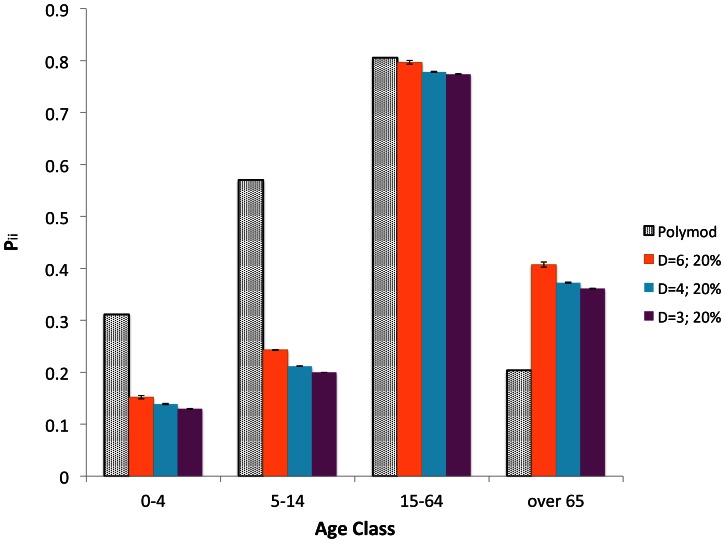
Proportion of contacts with individuals of the same age, 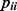. Comparison between the Polymod data and the simulated data on lattices with different dimensions (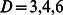).

We also evaluate the matrix 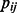, whose element gives the fraction of the contacts of the age-class *j* with individuals belonging to the age-class *i*, i.e. 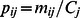. The information about the assortativity is encoded in the diagonal elements of this matrix: the higher is the value of 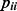, the higher is the assortativity of the age-class *i*. In Fig. 2, the values of the diagonal elements 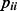 are compared to those obtained from the Polymod data. One can see that the agreement between the numerical and experimental data, with respect to the assortativity, is not satisfactory. In other words, the model reproduces correctly the total daily contact numbers in each age-class 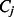, but not the mixing patterns 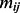 among the age-classes. We will discuss further this limit in the Discussion session.

Comparing different lattice topologies, one can notice that, varying the dimension of the lattice, different contact patterns are obtained with slightly different assortativity. The agreement with the Polymod data is slightly better in the 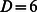 lattice than in the other dimensions. This is true in each age-class except for elderly people (over 65 years old). However, we expect this discrepancy to be not relevant here, since elderly people have been only marginally involved into the spreading of the H1N1 influenza.

### Epidemics Curves

In this section, the model is tested for the Italian epidemiological data of the H1N1 pandemic during the season 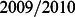. We assume both the infective period, 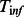, and the stop time, 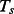, to follow exponential distributions. The average infective period is set equal to 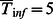 days, which corresponds to the typical duration of influenza symptoms (with a truncation of the distribution at 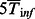). The average stop time is set equal to 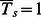 day, which corresponds to the typical duration of the asymptomatic phase for the influenza (with a truncation of the distribution at 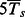).

On each lattice, we set the parameters 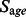 (age-dependent susceptibility) in order to reproduce the peak of the outbreak in each age-class. The simulations are initialized with a density of infected individuals, randomly distributed on the lattice, which is equal to the 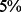 of the density of infected individuals observed at 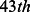 week in the Italian epidemiological data of the H1N1 pandemic.

**Figure 3:**
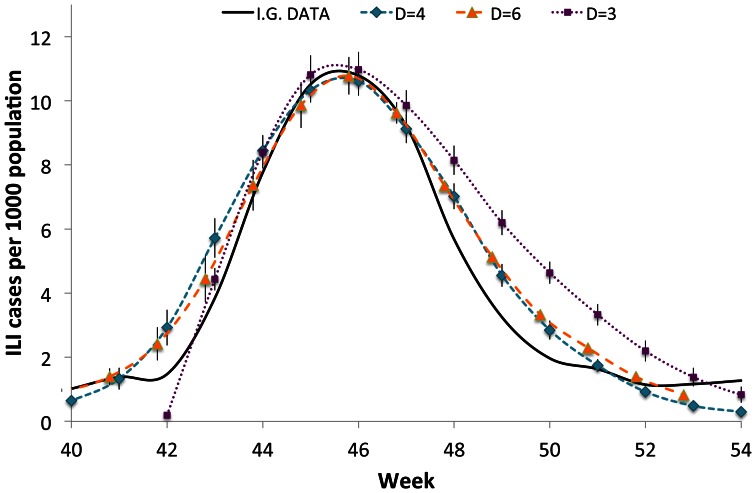
ILI cases of the H1N1 pandemic in Italy during the season 2009/2010. Comparison between the epidemiological data, furnished by the Italian Government, and the simulated data on lattices with different dimensions (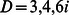).

In Fig. 3, we compare the overall observed illness prevalence (per thousand of individuals) of H1N1 influenza cases in Italy from the 40-th week of the year 2009, to the numerical data obtained in our model, simulated on lattices of different dimensions. The simulated illness cases and their errors are evaluated respectively as mean values and standard deviations over 32 independent processes.

Comparing the slopes of the epidemic curve in 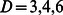 from 42-th week to 43-th week, one can see that at the beginning of the outbreak, the slope in 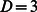 is higher than in 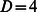 and 6. This circumstance is consistent with the fact that, performing a random distribution of the initial infected individuals, the probability to have two initial infected individuals that are nearest neighbors to each other (which corresponds to an “inefficient” distribution of initial cases) increases with the dimension of the lattice. After the peak, the process turns off more slowly in lattices with small *D* rather than in higher dimensional cases. From Fig. 3, one can also see that the agreement between experimental and numerical data is slightly better for 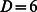, which is also the case that has the highest values of the assortativity among those considered in the present paper. Thus in the following we will concentrate on this case.

**Figure 4:**
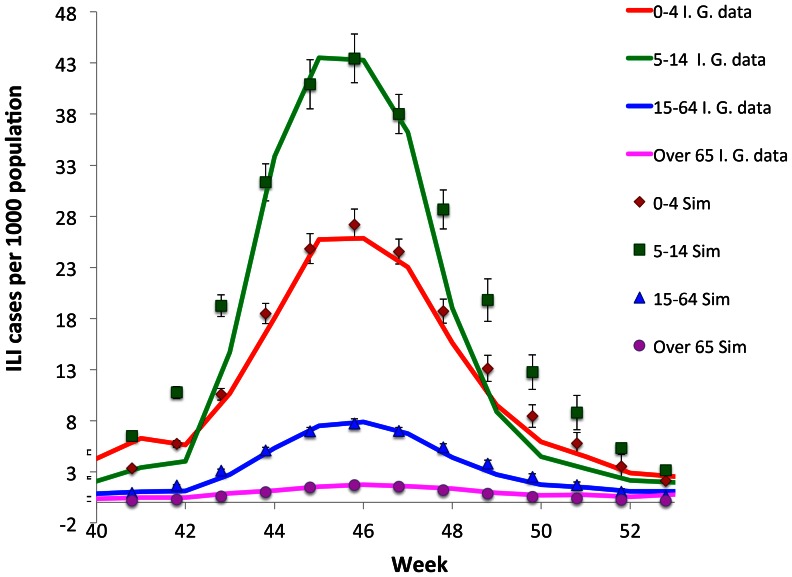
Age distribution of the ILI cases of the H1N1 pandemic in Italy during the season 2009/2010. Comparison between the epidemiological data, furnished by the Italian Government, and the simulated data on the *D* = 6 lattice.

In Fig. 4 we compare the simulated illness cases by age group, on the 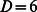 lattice, with the corresponding data on the H1N1 influenza cases in Italy, furnished by the Italian Government. The dissimilarity among different age-class susceptibilities indicated in Table 3, reflects the fact that the H1N1 virus had different incidence on different age classes, causing symptomatic disease mainly in younger population, as a consequence of a pre-existing partial immunity of older people [15]. The smaller incidence on the 0–4 years old with respect to the 5–14 years old, may be due to the minor exposure of this age-class, because of the small percentage of children, which attend day nursery in Italy.

**Table 3:** Susceptibilities.

age-class	*S_age_*
0–4	0.07
5–14	0.12
15–64	0.019
+65	0.004

Age dependent susceptibilities for the simulation on the 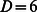 lattice.

The comparison between observed and simulated data in Fig. 4 shows that our model reproduces the epidemiological data during the epidemic peak (from week 44-th to week 47-th) for all the age-classes. For age groups 0–4, 15–64 and +65 the model reproduces quite accurately the entire epidemic evolution. However, it fails to reproduce the very beginning of the outbreak, as well as the descendant phase of the epidemic, for the 5–14 years old age group. As observed in the previous sections, although our model well reproduces the diagonal element 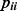 of the contact matrix of the Polymod project in the adult case, a worse agreement is observed for the other age-classes (particularly evident for the age-class 5–14), which may reflect in the mismatch observed here between the numerical and the epidemiological data.

As discussed below, other reasons could also contribute to this disagreement. One should first observe that the Italian Government data we are comparing with, are those relative to the illness consultations and not to the ascertained cases. A direct and more reliable measure of the incidence of infection in the population would have been obtained by comparing the presence of antibodies in serum samples before and after the pandemic (e.g. [44]). However, in our knowledge such kind of analysis has not been performed for the Italian case and thus age-classes immunity data are not available. Moreover, also pharyngeal swabs on ILI cases have not been performed systematically, so that a reliable statistic on positive cases is not available. Illness consultations are of course a much more reliable estimate of the real epidemic diffusion during the epidemic peak (when the alert of population and public health system is utmost) but they are less significant at the beginning and at the end of the epidemic. Therefore, part of the disagreement at the beginning of the outbreak for the age-class 5–14 (that is the one mostly involved into the epidemic) may be due to an underestimation of the influenza diffusion at that time, since not all the cases of influenza like symptoms led to illness consultations, as instead it mostly happened during the peak. Part of the disagreement in the descendant phase for the age group 5–14 may be instead due to self-initiated health care measures carried out by many Italian families. Indeed, even if no closure of schools was decided by the Italian Government, the spread of fear during the peak (also due to a strong media campaign), induced many families to keep children at home, drastically reducing their scholar and extra scholar activities during that period.

### Estimation of Epidemiological Parameters

The estimation of the reproductive number, *R*, and the generation time interval, *v*, leads to some interesting results.

**Figure 5:**
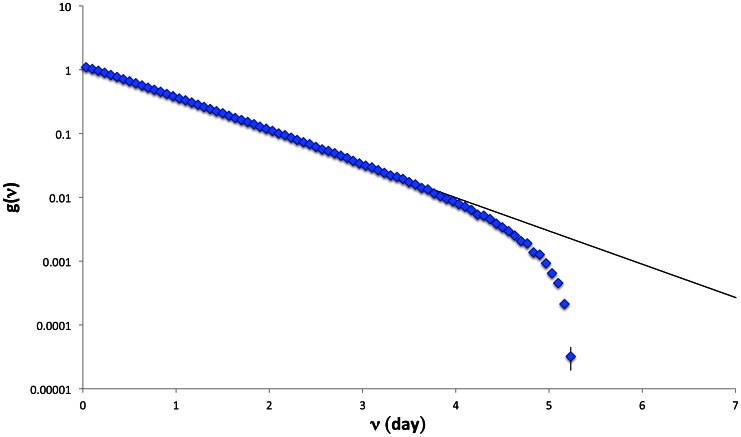
Generation time distribution, 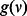, obtained with the simulations on the 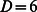 lattice.

For the generation time, *v*, we adopt the backward definition, defining it as the time interval between the infection of an individual and that of her/his infector. The generation time distribution, 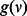, obtained in the Experiment 3, is shown in Fig. 5. From the generation time distribution, we evaluate the mean generation time, which turns out to be 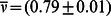 day. Such low value is mainly due to the absence of a latent period, and to the fact that, in our model, the contagions happen essentially during the dynamical phase (i.e. before the recovering at home of the infected individual).

As we see in Fig. 5, 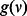 is well fitted by an exponential function, 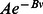, with parameters 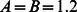. Deviation from this functional form is observed at large *v*. It is worth noticing that the fitting parameters *A* and *B* exactly coincide with the quantity 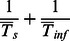, where 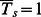 day and 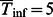 days are respectively the average stop time and the average infective period adopted in our simulations. Therefore, 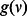 can be factorized as
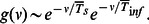
(8)


The functional form obtained in Eq. (8) for the generation time distribution can be interpreted in the following way [37]: 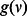 may be seen as the product of 3 distinct probabilities
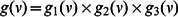
(9)where 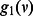, is the probability that an individual infected at time 0 is still infective at time *v*, 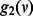 is the probability that an individual infected at time 0 meets a susceptible at time *v*, and 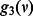 is the probability of contagion. Adopting the parallelism with the demographic process, i.e. treating the infection process as a birth process, Eq. (9) expresses the probability of a birth from a mother of age *v* (i.e. 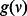) as the product of the survival probability of a mother of age *v* (i.e. 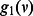), times the fecundity function (i.e. 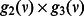), here factorized as the probability of an useful reproductive contact, times the probability of conception.

The function 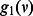 in Eq. (9) coincides with the probability that the infective period of the infector is greater than the actual time, *v*, and, it is by definition the complementary cumulative distribution function of the infective period, evaluated at time *v*:
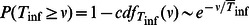
(10)


The function 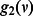 is proportional to the probability that the potential infector is not in the stop (i.e. symptomatic) phase, which is in turn given by the probability that the stop time of the infector is greater than the actual time *v*:
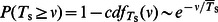
(11)


Finally, 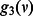 is simply given by the weighted average of the susceptibility 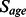 on the age groups and does not depend on *v*. By replacing 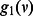 and 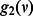 in Eq. (9) respectively with Eq. (10) and Eq. (11), Eq. (8) is obtained. The deviation of 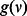 from this functional form, for values of 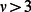, is therefore due to the truncation of both the stop time and infective period distributions at 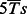. This is an interesting result, which gives an explicit connection among the distributions of the generation time, the stop time and the infective period.

The reproductive number *R*, which is the mean number of secondary cases generated by a typical single infected case in a population of entirely susceptible individuals, is related to the generation time distribution 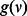, being the inverse of the Laplace transform of such distribution [45]

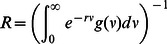
(12)where the parameter *r* is the cumulative exponential growth rate, and can be derived by exponential regression of the stabilized cumulative number of cases. In our simulations, we measure *R* as [45]

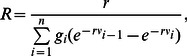
(13)where 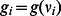 are the simulated frequencies of the generation time within 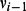 and 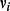. We evaluate r in a time window of one week at the beginning of the simulation, obtaining 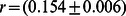 day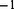 which corresponds to a reproductive number 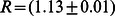. This is consistent with what found in recent literature for other countries, which indicates modest values for R [46], [47].

## Discussion

In this paper, we propose a very simple model for the epidemic spreading in an age-structured population with dynamic contacts. In spite of its simplicity, the model has some “good” properties: it is a dynamic contact network, in which individuals have a variable number of contacts with variable identities; it includes disease adaptive mobility restrictions; few demographic information is required. The model correctly reproduces the age specific epidemic curves of the H1N1 pandemic in Italy for each age-class, except young people (5–14 years old). For this age group, the agreement between the numerical and epidemiological data is only found at the peak of the epidemic (from 44*th* week to 47*th* week).

The epidemiological model can be immediately extended in order to include a latent period, a susceptible-infector dependent transmission rate, a variable infectivity and self-initiated behavioral conditioning.

The application of intervention strategies such as vaccination or reduction of mobility can be easily implemented into the model. For example, one can study the effect of vaccination by introducing a certain percentage of immune individuals at a certain time step. Simulations show that a vaccination campaign of the 20 percent of the Italian population with a vaccine effectiveness of 70 percent at 46*th* week produces a reduction of the cumulative attack rate of the 37 percent, and a reduction of the 67 percent if the measure becomes effective at 44*th* week.

There are some limitations which affect this study, concerning both the dynamic model and the transmission model.

First, the total daily contact numbers 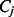, obtained with the mobility rules discussed in the Results Section, are in good agreement with the Polymod data, but the assortativity of Polymod is not properly reproduced, as shown in Fig. 2. This is a limit of our study and is related to the double role of the parameter 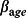: on one hand, it works as a regulator of the assortativity and, on the other hand, it regulates the overall number of contacts. In particular, the high assortativity regime is obtained with 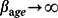 but, in this limit, the mobility of individuals strongly reduces. The 5–14 age-class has the peculiarity to be at the same time, the most assortative and the most dynamic age-class. For this reason, it is hard to reproduce these two features within only one parameter.

An interesting question is how the results would change if the 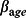 parameters were chosen in order to fit the number of internal contacts 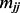 per unit time, rather than the total number of contacts 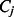, for each age class *j*. One should first observe that this alternative strategy would not automatically lead to an improvement of the overall assortativity, which indeed depends on the ratio among the internal and the total contacts. In particular, with this alternative procedure, there would be no constraints on the total number of contacts (unless introducing other fitting parameters to fix them) with the result that the total contacts could assume values very different from the Polymod data. We performed a set of such simulations and found an improvement in the assortativity of young people, and a worsening in that of adults and elderly people. However, no significant improvement of the agreement between the epidemiological data and the simulated ones were observed in this different setup, giving the two procedures essentially similar results. In our opinion, to improve the agreement with the epidemiological data, one should construct a dynamic model that is able to reproduce the entire matrix of mixing patterns rather than only some of its entries.

A different strategy could be to consider more complex topologies, in which individuals of different age-classes live on lattices of different dimensions. Another possibility that we are exploring is the introduction of kinetic constraints or repulsive interactions among individuals of different age-classes, that we will discuss in a future work.

The simplifications adopted in the transmission model are numerous. First, we do not consider the time of exposure to the infection. Therefore, short episodes of contacts are on the same footing as long standing contacts. This simplification is quite reasonable for viral diseases, as influenza, for which even short contacts can be sufficient for the transmission process. For other kind of infections, as the bacterial ones, the time of exposure is relevant for the transmission and thus one should properly include it in the model. Moreover, in the present study, the infectivity of the individuals is assumed to be constant during the entire infective period. In principle, one should consider the reduction of the viral load during the manifestation of the disease. There are examples in literature, in which the infectivity is a function of the time elapsed from the beginning of the infective period, as in Ref. [2], where the infectivity is assumed to follow a log-normal distribution. Similarly, we do not consider the effect of the simultaneous presence of more than one infected individual in the neighborhood of the susceptible one: the probability of an individual to be infected does not depend on the number of her/his nearest neighbors, which are simultaneously infected, but only on the age-class of the susceptible individual. Both these simplifications correspond to disregard the effect of the viral load in the infection process, which is indeed entirely ascribed to the immunological status of the susceptible individual. To disregard the viral load in the transmission process is acceptable for highly infective disease, as the influenza pandemic. However, the model can be easily “upgraded” in order to overcome all the previous limits, introducing a susceptible-infector dependent transmission rate, a variable infectivity, and to make the transmission rate dependent on the number of simultaneously infected nearest neighbors.

In conclusions, in this paper we present a very essential model focused on few ingredients (the age-class distribution, the dynamic nature of contacts, and the daily contact numbers), assumed to be relevant for the spreading of an infectious disease. Our model belongs to the class of epidemiological models, which adopt the social contact hypothesis as the leading key to interpret and reproduce the contagion process. Being in countertrend with respect to the mostly used approaches in recent literature (I.e. IBM and Metapopulation models), which involve highly detailed socio-demographic/mobility models and require an huge amount of input data, we believe that such a simple model could open a valuable alternative perspective with respect to the mostly used “realistic” epidemiological models.
